# Genome-Wide SNP Analysis for Milk Performance Traits in Indigenous Sheep: A Case Study in the Egyptian Barki Sheep

**DOI:** 10.3390/ani11061671

**Published:** 2021-06-03

**Authors:** Ibrahim Abousoliman, Henry Reyer, Michael Oster, Eduard Murani, Ismail Mohamed, Klaus Wimmers

**Affiliations:** 1 Leibniz Institute for Farm Animal Biology, Wilhelm-Stahl-Allee 2, 18196 Dummerstorf, Germany; abou-soliman@fbn-dummerstorf.de (I.A.); reyer@fbn-dummerstorf.de (H.R.); oster@fbn-dummerstorf.de (M.O.); murani@fbn-dummerstorf.de (E.M.); 2 Desert Research Center, Department of Animal and Poultry Breeding, 1 Mathaf El-Matareya St., El-Matareya, 11753 Cairo, Egypt; ssmm_ismail@yahoo.com; 3 Faculty of Agricultural and Environmental Sciences, University of Rostock, Justus-von-Liebig-Weg 7, 18059 Rostock, Germany

**Keywords:** Barki sheep, milk performance, genome wide SNPs, genomic regions, candidate genes

## Abstract

**Simple Summary:**

The Barki sheep is one of the three main breeds in Egypt, which is spread mainly throughout the northwestern coastal zone, which has harsh conditions. Considering the harsh, semi-arid habitat of this breed, milk performance traits such as milk yield and milk composition have a very important role in the feeding of newborn lambs and affect their growth during the early stage of life. In this study, rare milk performance data and genomic information of Barki sheep were used to uncover diversified genomic regions that could explain the variability of milk yield and milk quality traits in the studied population of Barki ewes. Genome-wide analysis identified genomic regions harboring interesting candidate genes such as *SLC5A8*, *NUB1*, *TBC1D1*, *KLF3* and *ABHD5* for milk yield and *PPARA* and *FBLN1* genes for milk quality traits. The findings offer valuable information for obtaining a better understanding of the genetics of milk performance traits and contribute to the genetic improvement of these traits in Barki sheep.

**Abstract:**

Sheep milk yield and milk composition traits play an important role in supplying newborn lambs with essential components such as amino acids, energy, vitamins and immune antibodies and are also of interest in terms of the nutritional value of the milk for human consumption. The aim of this study was to identify genomic regions and candidate genes for milk yield and milk composition traits through genome-wide SNP analyses between high and low performing ewes of the Egyptian Barki sheep breed, which is well adapted to the harsh conditions of North-East Africa. Therefore, out of a herd of 111 ewes of the Egyptian Barki sheep breed (IBD = 0.08), ewes representing extremes in milk yield and milk quality traits (*n* = 25 for each group of animals) were genotyped using the Illumina OvineSNP50 V2 BeadChip. The fixation index (F_ST_) for each SNP was calculated between the diversified groups. F_ST_ values were Z-transformed and used to identify putative SNPs for further analysis (Z(F_ST_) > 10). Genome-wide SNP analysis revealed genomic regions covering promising candidate genes related to milk performance traits such as *SLC5A8*, *NUB1*, *TBC1D1*, *KLF3* and *ABHD5* for milk yield and *PPARA* and *FBLN1* genes for milk quality trait. The results of this study may contribute to the genetic improvement of milk performance traits in Barki sheep breed and to the general understanding of the genetic contribution to variability in milk yield and quality traits.

## 1. Introduction

Sheep have been raised for milk production for thousands of years, before most other mammalian species [1]. In many countries around the world, especially in the Mediterranean region, sheep milk and its products are widely consumed by humans and are considered an important food resource. Sheep contribute about 5% of the total annual milk production in Egypt, whereas cows and buffaloes are the major suppliers of milk. This is a reflection of the management system of sheep milk production, which is characterized by subsistence and smallholder farming systems [2]. Sheep milk is highly similar to human milk in fatty acids composition, which makes it a suitable raw material for infant formula production [3]. Moreover, milk is the most important feed resource for newborn lambs during the early stage of their lifetime, from birth to weaning age (90 days), providing energy and proteins for growth and antibodies against infections and diseases [4]. Milk components such as fat, protein and lactose are important indicators of milk quality, which affects the growth and healthy feeding of the newborn lambs. Sheep milk production and composition are influenced by genetic and environmental factors. Estimates of heritability for milk yield, fat content and protein content in some sheep breeds such as Churra ewes were 0.32, 0.29 and 0.41, respectively [5,6]. The Barki sheep is one of the three most important breeds in Egypt, as it has adapted well to the harsh environmental conditions of Egypt’s northwestern coastal zone, where it is raised for meat, as its main product, and milk, as its by-product. The total population of Barki sheep is about 470,000 heads, which are owned by small holders [7]. The current Barki sheep breeding is characterized by a phenotypic selection approach considering mainly the number of offspring and the growth performance of lambs. In addition, the general health status is included, which enables ewes and lambs to cope with the harsh environmental conditions. Neither a structured breeding program nor a genetic selection program is applied. The amount of milk produced by Barki sheep in particular is low compared to the other native Egyptian breeds or worldwide breeds, possibly due to the absence of any attempts to perform phenotypic or genomic selection of milk performance traits in this breed. This low production affects lambs’ growth and viability and increases the percentage of the lambs lost due to inanition. It is also noticeable that the production of milk and its composition varies greatly between individuals in the Barki sheep breed, which is attributed to both genetic and environmental factors [8]. Therefore, it is feasible to study the differences between high and low productive individuals. The development of high-density SNP arrays and bioinformatics tools enables researchers to detect genomic regions that contribute to phenotypic variation in different livestock species, using different approaches based on linkage disequilibrium, allele frequency or haplotype characteristics [9]. To gain further knowledge about the genetic architecture, the fixation index (F_ST_) approach of Weir and Cockerham is a suitable method, also for small data sets, to uncover genomic differences between experimental populations or groups and detect genomic regions with divergent allelic frequencies indicating putative candidate genes [10,11]. In this context, several studies were performed using genome-wide SNP data and revealed some candidate genes for milk traits in dairy cattle [12,13,14], sheep [15,16] and goats [17,18]. Previously, F_ST_ approach was conducted to detect some candidate genes for productive and reproductive traits such as fertility in Egyptian native Rahmani sheep breed [19]. The aim of the current study is to explore genomic differences of Barki ewes divergent in milk performance traits, thereby identifying genomic regions and candidate genes related to milk yield and milk composition.

## 2. Materials and Methods

### 2.1. Animals and Phenotypes

The experiment was conducted in accordance with all ethical and animal welfare standards of the Desert Research Center, taking into account all regulations in compliance with the European Union Directive for the Protection of Experimental Animals (2010/63/EU). A population of 111 Egyptian Barki ewes aged between 4 and 5 years was kept in the farms of Desert Research Centre, Ministry of Agriculture, Egypt under an intensive system and housed in semi-open yards for one breeding season. All ewes in the study were sired by 10 rams. Throughout the experimental period, ewes were fed daily on a feed concentrate (0.75 kg per head) and clover hay (0.5 kg per head). Fresh water was available to sheep ad libitum. Ewes were in the same parity and lactation period. Milk yield was recorded from parturition for a period of 3 months by hand milking in the morning and evening. Daily milk yield was measured by summation of the morning and evening milking. Total milk yield was calculated by summation of the daily milk yields for 90 days. Milk was sampled and stored at −20 °C. Milk from mixed samples of morning and afternoon milk were analyzed for percentages of fat, protein, lactose, and total solids using milko-scan (130 A/SN Foss Electric, Hillerod, Denmark). For genetic analysis of milk traits, both milk yield and milk composition served as selection criteria. For milk yield, in total 50 ewes were selected from the two tails of the phenotypic distribution and divided into two subgroups (high milk yield—HMY represent top 25 animals and low milk yield—LMY represent bottom 25 animals), each representing the extreme phenotypes for the milk yield trait. For milk composition, the measured values for fat, protein, lactose and total solids were used for a principal component analysis to calculate animal-individual eigenvalues. Therefore, the phenotypic correlation matrix was used to compute principal components using R statistical software [20]. The first and second principal components explained about 59.7% and 19.3% of the phenotypic variance of the traits. The first principal component was considered for grouping of animals according to milk quality (Supplementary Figure S1). Ewes having extreme negative loadings on PC1 were considered as high milk quality (HMQ) animals (*n* = 25), whereas individuals with extreme positive loading on PC1 were assigned to the low milk quality (LMQ) group (*n* = 25). Student’s *t*-test was used to compute the differences between the group means using SPSS V20 (IBM, New York, NY, USA). Phenotypic Spearman correlation coefficients among milk performance traits and PC1 were calculated for all animals (*n* = 111).

### 2.2. Genotyping and Quality Control

DNA was extracted from blood samples, collected from the jugular vein of all ewes, using the G-spin Total DNA Extraction kit (iNtRON Biotechnology, Seoul, Korea) according to the manufacturer’s instructions. Out of the entire population of 111 animals, 71 ewes were genotyped using the Illumina OvineSNP50 V2 BeadChip (Illumina, San Diego, CA, USA). The genetic relatedness of all pairs of ewes was assessed by calculating relative identity–by-descent (IBD) probabilities, which revealed an average relatedness of 0.08. The raw signal intensities of the 53,516 SNPs on the chip were imaged using the iScan Reader (Illumina). The signals were converted into genotype calls using the Genome Studio software (version 2.0). The SNPs with genotype call rates <90%, minor allele frequencies (MAF) <0.03 [21] and significant deviation from Hardy–Weinberg equilibrium at *p* < 10^−6^ were removed from analysis using JMP Genomics software (version 9). Base pair positions and names of SNP markers were updated to the latest version of the ovine genome of Texel breed (Oar_v3.1 accessed on 6 July 2020). SNPs not located on autosomes and lacking rs identifiers were excluded. After quality control, 49,184 SNPs were used for analyses.

### 2.3. Genome Wide F_ST_ Calculation

SNPRelate R package was used to calculate the F_ST_ of Weir and Cockerham for each SNP between the subgroups (LMY-HMY and LMQ-HMQ) [22]. The resultant distribution of F_ST_ values were Z-transformed and the extreme tail of the distribution was used to identify putative SNPs for further analysis, using a threshold Z(F_ST_) > 10. In addition, all SNPs that passed the cutoff threshold at Z(F_ST_) > 5 were listed in Supplementary Tables S1 and S2 [23]. Manhattan plots of the genome-wide Z(F_ST_) values were performed using qqman package in R software. Genomic regions with the highest Z(F_ST_) values were considered as region of interest. Genes within 1 megabase (Mb) regions up- and downstream of SNPs with highest Z(F_ST_) values were scrutinized based on positional and functional evidences according to the Ensembl database. Genes harboring a highlighted SNP were considered positional candidate genes. Genes within the 2-Mb window were considered functional candidate genes, taking into account their functional relationship to phenotypes using available gene annotations from the GeneCards (http://www.genecards.org (accessed on: 3 February 2021) and Uniprot (http://www.uniprot.org (accessed on: 3 February 2021) databases.

## 3. Results

### 3.1. Phenotypic Data of Milk Performance Traits

Descriptive statistics of milk yield and milk composition, comprising fat, protein, lactose and total solids percentages and principal component 1 (PC1) for milk quality (MQ), are shown for the Barki subgroups in Table 1. A high MQ is indicated by negative loadings on PC1, whereas a low MQ is represented by positive values.

Significant correlation coefficients were determined between the milk composition traits (Table 2). The highest correlation coefficient was obtained for TS and P (0.83), followed by the coefficients of F and TS (0.47), and TS and L (0.43). There was no considerable correlation between milk yield and milk composition traits. The correlation coefficients obtained for PC1 showed that all milk composition traits are correlated to varying degrees by PC1, with protein and total solids having the highest correlation coefficients.

### 3.2. Detection of Genomic Regions and Candidate Genes

**Figure 1:**
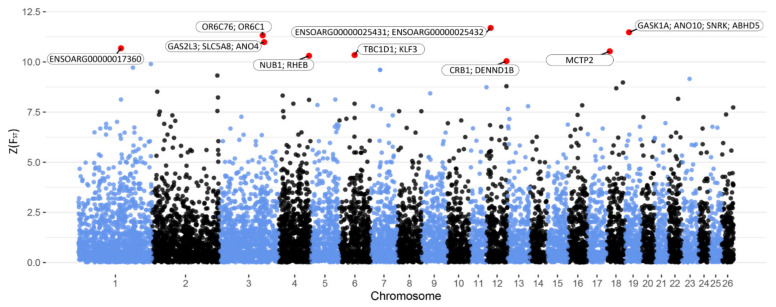
Manhattan plot of Z(F_ST_) values at each SNP for milk yield. The red dots represent the SNPs that passed the cut-off threshold at Z(F_ST_) = 10 and are labelled with candidate genes within a 2 Mb window.

The animals were divided into two subgroups representing extreme phenotypes for milk yield (HMY and LMY) and milk composition (HMQ and LMQ). Z(F_ST_) values were calculated to investigate the genomic differences between the groups using a genome-wide SNP panel. For milk yield, a number of genomic regions and SNPs were indicated to differentiate between the groups (Z(F_ST_) > 10, Figure 1). These regions and SNPs were distributed on the chromosomes 1, 3, 4, 6, 12, 18 and 19 (Table 3). Within these genomic regions, *OR6C75*, *ANO4*, *MCTP2* and *SNRK* were identified as positional candidate genes. Moreover, *SLC5A8*, *NUB1*, *TBC1D1*, *KLF3* and *ABHD5* were proposed as functional candidate genes, which are known to affect lactation, mammary gland development and secretion and fatty acids’ synthesis and lipids’ metabolism.

**Figure 2:**
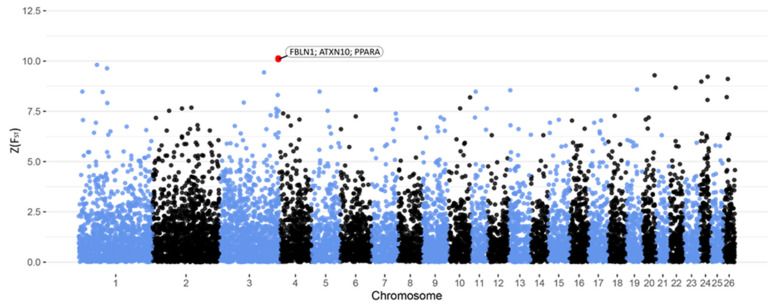
Manhattan plot of Z(F_ST_) values at each SNP comparing LMQ and HMQ Barki ewes. The red dots represent the SNPs that passed the cut-off threshold at Z(F_ST_) = 10 and are labelled with candidate genes within a 2 Mb window.

Figure 2 shows the Manhattan plot representation of SNP-specific Z(F_ST_) values for milk quality. A genomic region and corresponding SNPs located on chromosome 3 are highlighted to be linked to this trait in the Barki sheep population (Table 3). Positional and functional candidate genes derived by the selected SNPs are indicated in Table 3. Within the genomic region on chromosome 3, *ATXN10* gene was identified as a positional candidate gene. Moreover, *FBLN1* and *PPARA* genes were designated as functional candidate genes in the identified genomic region.

## 4. Discussion

The averages of milk yield and milk composition traits (fat, protein, lactose and total solids percentages) in this study were similar to the previously recorded values of Barki ewes with 44.7 kg, 4.17%, 3.34%, 5.01% and 15.88%, respectively [24]. The results of the correlation analysis between the recorded milk traits confirmed the positive correlation among milk composition traits [25]. In contrast to other studies in sheep and cattle, there was no considerable negative correlation between milk yield and milk composition traits, possibly due to the overall low milk production of Barki sheep and the limited breeding efforts on these traits. Furthermore, a positive correlation was revealed between PC1 and milk composition traits as shown in Table 2. For comparison, the average milk yield of the Rahmani breed (70.75 kg), which is another important indigenous Egyptian sheep breed, was reported to be significantly higher [26]. The correlation results among milk yield and milk components were in agreement with those of ewes from the ancient Iberian Churra breed, which also have low average milk performance [6]. The ewes in this study were considered not substantially related according to genetic relatedness (IBD = 0.08) and were suitable for the application of the F_ST_ approach [27]. For milk yield, a total of seven genomic regions were identified to differentiate comparing HMY and LMY animals as shown in Table 3. Scrutiny of the genes in the identified genomic regions revealed functional candidates on chromosomes 3, 4, 6, 18 and 19. Several QTL for milk yield on these chromosomes in different genomic regions in the Sheep Genome were reported previously in various sheep breeds [8,28,29,30,31,32,33]. The same genomic region on chromosome 18 was detected to be associated with milk yield in East Friesian and Dorset sheep breeds [32]. In the genomic region on chromosome 3 at 169.8 Mb, Solute Carrier Family 5 (Sodium/Monocarboxylate Cotransporter) Member 8 (*SLC5A8*) was previously reported to be associated with milk yield in Italian Holstein dairy cows [34]. The genomic region on chromosome 4 harbors the Negative Regulator of Ubiquitin-Like Proteins 1 (*NUB1*) gene as one of the proposed genes affecting milk yield and contributing to the proteasomal degradation pathway. *NUB1* was previously proposed as a strong candidate gene explaining the variation in milk yield in Gir × Holstein (Girolando) crossbreed animals [35]. The QTL on chromosome 6 at 57 Mb includes *TBC1* Domain Family Member 1 (*TBC1D1*) and Kruppel-Like Factor 3 (*KLF3*). Selection signatures study in dairy and beef cattle revealed *TBC1D1* as candidate for milk production [36]. In Holstein cows, a scan for polymorphisms in *TBCID1* yielded two SNPs associated with milk protein yield [37] and another SNP associated with fat and protein percentages [38]. The importance of *KLF3* was suggested in Chinese Holstein cows based on its physiological and biochemical functions in many processes such as cell proliferation, differentiation, homeostasis and apoptosis [39,40]. Moreover, a SNP in *KLF3* was significantly associated with milk yield and protein yield also in Chinese Holstein [41]. The Abhydrolase Domain Containing 5 (*ABHD5*) gene, which resides on chromosome 19 at 15.5 Mb, represents a prospective functional candidate, based on its important role in lipid metabolism, the energy balance signaling pathway and triglyceride metabolism in dairy cows and Qinchuan cattle [42,43].

For milk quality, a genomic region on chromosome 3 was shown to be differentiated between HMQ and LMQ ewes, confirming previously reported QTL for milk fat percentage [30,33], protein percentage [44,45] and lactose percentage [46]. Within this genomic region on chromosome 3, *PPARA* and *FBLN1* genes were proposed as candidates. The Peroxisome Proliferator Activated Receptor Alpha (*PPARA*) gene located at 220.6 Mb is a member of the *PPAR*s family, which has a critical role in the regulation of milk fat synthesis in lactating ruminants [47]. *PPARA* is one of the genes involved in lipid metabolism in mammary gland in dairy cows [48]. In Charolais × German Holstein cross-breed dairy cows, *PPARA* was associated with milk yield and protein synthesis [49]. In line with the results of the Barki study, Fibulin 1 (*FBLN1*) located on chromosome 3 at 220 Mb was reported to be associated with milk protein yield and protein percentage in dairy cattle [25]. In addition, *FBLN1* was reported to play a critical role in the development and cell differentiation of the mammary gland [50]. However, due to the limited sample size available for Barki sheep in the current study, the results deserve further investigation involving a larger number of animals and other indigenous sheep breeds.

## 5. Conclusions

The results of the genome-wide analysis uncovered some genomic regions contributing to variability in milk performance traits such as milk yield and milk quality in Bakri sheep. These regions harbor some interesting functional candidate genes such as *SLC5A8*, *NUB1*, *TBC1D1*, *KLF3* and *ABHD5* for milk yield, and *PPARA* and *FBLN1* for milk quality traits. These genes deserve further investigation to analyze the association between genetic variations of these genes and their respective milk phenotypes. Given the current absence of structured genetic improvement programs in Barki sheep, the current analysis provides insights into genomic regions that are critical for milk quantity and quality in ruminants. Our findings offer valuable information for the future improvement of milk performance traits and the associated assurance of offspring supply in the Barki sheep breed.

## Figures and Tables

**Table 1:** Descriptive statistics of milk performance traits.

Trait	Group *	N	Mean	SE	Min	Max	*p* Value **
Milk yield MY (kg)	HMY	25	41.97	2.02	31.50	72.00	*p* < 0.001
	LMY	25	17.20	0.71	9.90	23.40	
Milk quality (MQ; PC1)	HMQ	25	−1.11	0.08	−0.63	−2.21	*p* < 0.001
	LMQ	25	2.05	0.29	4.91	0.14	
Milk fat (%)	HMQ	25	6.28	0.26	4.78	9.60	*p* < 0.001
	LMQ	25	2.61	0.13	1.45	3.60	
Milk protein (%)	HMQ	25	6.85	0.25	5.20	9.20	*p* < 0.001
	LMQ	25	4.09	0.10	2.85	4.60	
Milk lactose (%)	HMQ	25	7.99	0.14	7.30	9.90	*p* < 0.001
	LMQ	25	5.14	0.22	1.01	6.10	
Total solids (%)	HMQ	25	25.49	0.87	20.00	33.10	*p* < 0.001
	LMQ	25	14.83	0.18	12.68	16.24	

* HMY = High milk yield, LMY = Low milk yield, HMQ = High milk quality, LMQ = Low milk quality. ** *p* value computed using *t*-test, SE = Standard error, PC1 = Principal component 1.

**Table 2:** Phenotypic Spearman correlation among milk performance traits.

Trait	MY	Fat	Protein	Lactose	Total Solids	PC1
MY	1					
Fat	−0.12	1				
Protein	−0.05	0.38 **	1			
Lactose	0.05	0.26 **	0.29 **	1		
Total Solids	0.06	0.47 **	0.83 **	0.43 **	1	
PC1	−0.06	0.69 **	0.90 **	0.61 **	0.94 **	1

** Highly significant correlation (*p* < 0.01) using *t*-test for the significance. PC1 = Principal component 1.

**Table 3:** Genomic positions and putative candidate genes derived from SNPs differentiating between ewes divergent in milk yield and milk quality (Z(F_ST_) > 10).

Trait	Rs Name	Chr	Position	MAF	F_ST_	Z(F_ST_)	Candidate Genes *
Milk yield	rs412092721	1	158753375	0.477	0.263	10.68	*ENSOARG00000017360*
	rs428217479	3	164177406	0.429	0.278	11.32	*OR6C76*, *OR6C1*, *OR6C75*
	rs430736025	3	169823214	0.374	0.270	10.99	*GAS2L3*, *SLC5A8*, *ANO4*
	rs420351948	4	109338529	0.332	0.319	13.07	*ENSOARG00000001351*
	rs399050266	4	113362135	0.350	0.254	10.30	*NUB1*, *RHEB*
	rs418394216	6	57451934	0.201	0.255	10.34	*TBC1D1*, *KLF3*
	rs427343726	12	15424350	0.228	0.287	11.70	*ENSOARG00000025431*, *ENSOARG00000025432*
	rs412626910	12	79966574	0.421	0.247	10.04	*CRB1*, *DENND1B*
	rs430297634	18	11841541	0.433	0.259	10.53	*MCTP2*
	rs423654488	19	15202234	0.352	0.281	11.48	*GASK1A*, *ANO10*, *SNRK*-*ABHD5*
Milk quality	rs408700818	3	220103217	0.370	0.300	10.14	*ATXN10*, *FBLN1*, *PPARA*
	rs414244120	3	220048441	0.485	0.299	10.09	*FBLN1*, *ATXN10*, *PPARA*

* Gene names in bold = functional candidate genes; underlined = positional candidate genes; only *italic* = closest up- and downstream located genes within 1 Mb window; Chr = Chromosome, MAF = Minor allele frequency.

## Data Availability

The data presented in this study are available on request from the corresponding author.

## Supplementary Materials

The following are available online at https://www.mdpi.com/article/10.3390/ani11061671/s1. Table S1: Genomic positions derived from SNPs differentiating between ewes divergent in milk yield (Z(F_ST_) > 5), Table S2: Genomic positions derived from SNPs differentiating between ewes divergent in milk quality (Z(F_ST_) > 5), Figure S1: Eigenvalues from the principle component analysis for milk quality traits. Animals have been assigned to low (red) and high (blue) milk quality.
