# Supplementary material for: Genome-Wide SNP Analysis for Milk Performance Traits in Indigenous Sheep: A Case Study in the Egyptian Barki Sheep
Source: Animals (Basel). 2021 Jun 3;11(6):1671. doi: 10.3390/ani11061671 (PMC8228706; doi:10.3390/ani11061671)
Supplement: Supplementary file 1 [file animals-11-01671-s001.zip › animals-1219182 supplementary figureS1.pdf]

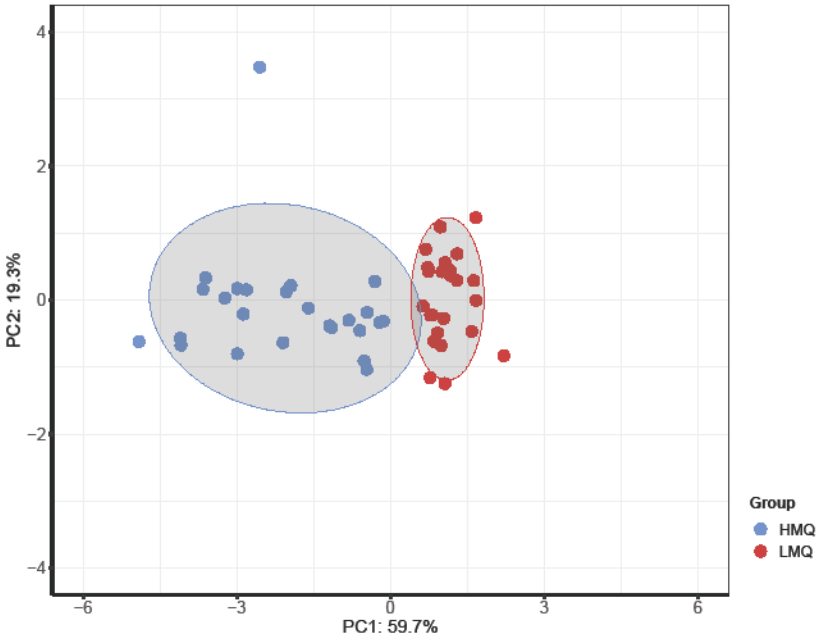

Figure S1. Eigenvalues from the principle component analysis for milk quality traits. Animals have been assigned to low (red) and high (blue) milk quality. HMQ- high milk quality; LMQ- low milk quality.
